# Comparative Study of the Management of Distal Tibia Fractures by Nailing Versus Plating

**DOI:** 10.7759/cureus.48321

**Published:** 2023-11-05

**Authors:** Rohit Nath, Saurabh Saxena, Chetan Singh, Chandan Kumar, Santosh Kumar Singh

**Affiliations:** 1 Orthopaedics, Ganesh Shankar Vidyarthi Memorial Medical College Kanpur, Kanpur, IND; 2 Orthopaedics and Traumatology, Maa Vindhyavasini Autonomous State Medical College, Mirzapur, IND

**Keywords:** wound complications, aofas score, intramedullary nailing, locking plating, distal tibia fracture

## Abstract

Introduction

The purpose of this study was to evaluate the management of distal tibial fractures treated by interlocking nail and plate osteosynthesis and to assess their functional outcome according to the American Orthopaedic Foot and Ankle Society (AOFAS) score and complications.

Methods

Twenty patients were operated on in each group, i.e., intramedullary nailing (IMN) and plating (minimally invasive plate osteosynthesis, MIPO). The patients were regularly followed up at six weeks, 12 weeks, six months, and one year and evaluated clinically and radiologically with respect to operating time, union time, and functional outcome on the basis of AOFAS score and complications.

Results

The mean union time for the IMN group was 18.45±2.45 weeks and for the MIPO group was 20±3.21 weeks (p-value >0.05). The mean AOFAS score in the MIPO group was 91.2±6.81 and in the IMN group was 92.6±5.41 (p-value >0.05). Lesser complications in terms of implant irritation, ankle stiffness, and infection were observed in the IMN group than in the MIPO group (p-value <0.05).

Conclusion

Both the IMN and MIPO groups had satisfactory outcomes for treating distal tibial fractures, with a higher risk of wound complications in the MIPO group.

## Introduction

The incidence of distal tibial fracture is 0.6% and constitutes approximately 10%-13% of all tibial fractures [1]. Distal tibial fractures are usually associated with significant soft tissue injury, and if they are not treated properly, they can cause significant long-term morbidity. Precarious blood supply and lack of adequate muscle coverage make the patient prone to a gamut of complications such as delayed union, non-union, and wound-related complications.

The distal tibial metaphysis is drawn by constructing a square with its side defined by the widest portion of the tibial plafond [2].

Currently, both minimally invasive plate osteosynthesis (MIPO) and intramedullary nailing (IMN) are established procedures with their pros and cons. Nailing is more frequently associated with knee pain and malalignment [3,4], whereas plating results in more wound problems and implant prominence [5].

The American Orthopaedic Foot and Ankle Society (AOFAS) ankle-hind foot score is a commonly used clinical rating system to assess ankle and hind foot function. The scale consists of a 100-point scoring system with three main components: pain, function, and alignment.

The purpose of this study was to compare the outcomes of management of extra-articular distal tibial fractures treated by MIPO and IMN based on clinical and functional outcomes (AOFAS score) and radiological outcomes by serial radiographs and complications.

## Materials and methods

This was a prospective cohort study conducted on 40 consecutive patients with extra-articular distal tibial fractures between September 2018 and September 2020. Written informed consent was obtained, and the patients were enrolled after clearance by the institutional ethics committee. Patients were allocated to either the IMN (group one) or the MIPO group (group two) based on the preference of the treating surgeon.

The inclusion criteria were patients with fractures of the distal end of the tibia with no articular incongruity (AO/OTA 43 A), fractures less than three weeks old at the time of surgery, and skeletally mature patients (>18 years). Patients with pathological fractures, fractures with intra-articular extension, associated malleolar fractures or ankle dislocations, open fractures, any prior pathology in the ankle joint, segmental tibial fractures, patients with neurovascular injury, patients who were medically unfit or not willing for surgery, or any other fracture in the ipsilateral extremity were excluded from the study.

Patients were compared with respect to the AOFAS score (primary outcome measure), surgical time, complication rate, and union time (secondary outcome measures).

After stabilisation and splinting the fracture in the emergency, the patients were radiologically investigated by traction X-ray of the affected ankle with leg anterior-posterior (AP) and lateral (LAT) views and routine haematological work-up. CT scan was obtained in some patients to rule out intra-articular extension. Patients with gross swelling were splinted until swelling subsided and operated after the appearance of wrinkle sign.

Surgical technique

Fixation by Intramedullary Nail

Patients were laid supine on the operating table, and the limb was prepped and draped up to the mid-thigh. Fibula fracture, if present in the distal one-third, was fixed either with a plate or an intramedullary titanium elastic nail based on the preference of the treating surgeon. The knee was flexed to 90 degrees and the leg was suspended by the edge of the table. A 5 cm midline incision was made over the ligamentum patellae and retracted laterally. The entry point was on the anterior edge of the tibial plateau and was centered over the medullary canal in the AP view. Entry was made with a curved awl. The fracture was reduced, and a guide wire was passed across the fracture site.

If closed reduction was unsuccessful, a reduction clamp was applied percutaneously or with a small incision. Reaming of the medullary canal was performed sequentially up to an appropriate size. In some cases, a blocking screw (poller screw) was placed in the distal fragment to guide the nail and correct the fracture deformity. A tibial intramedullary interlocking nail was inserted over the guide wire under image intensifier control. Three distal locking and two proximal locking were performed.

Fixation by Distal Tibial Locking Plate

The patient was placed on a radiolucent operating table in the supine position and a tourniquet was applied. Pressure was set according to the systolic blood pressure. The leg was prepped and draped up to the mid-thigh. The length of the plate was estimated by placing it on the skin while checking its position using fluoroscopy. A precontoured locking distal tibia plate of appropriate length was selected depending on the fracture geometry. A 3 to 4 cm anteromedial incision was made at the tip of the medial malleolus, and the plate was subcutaneously slid proximally along the medial surface of the tibia after making a tunnel with a tunneling device. After reducing the fracture closed or by percutaneous reduction manoeuvres, the plate was fixed with at least four locking screws distally and three locking screws proximally. Fixation of the fibula was performed at the discretion of the treating surgeon. After that, the wound was closed in layers and the dressing was applied.

Follow-up and rehabilitation protocol

All patients were followed up in OPD at six weeks, 12 weeks, six months, and one year. We allowed partial weight bearing only after signs of union in the form of bridging callus on at least three cortices out of four on radiograph and clinically as the absence of tenderness and movements at the fracture site. At each visit, radiographic evaluation was performed by taking radiographs of the ankle with leg anteroposterior and lateral views to check for alignment. Signs of radiographic union and functional evaluation were performed using the AOFAS score [6].

Statistical analysis

Quantitative data were compared using Student’s t-test, and qualitative data were analysed using the chi-square test. All statistical calculations were performed using SPSS version 16 (Chicago, IL, USA), and the level of statistical significance was set at 0.05.

## Results

A total of 20 patients were operated in each group i.e. IMN group and MIPO group. Five patients in the IMN group and three patients in the MIPO group did not meet the inclusion criteria and were therefore excluded from the study. There were 13 males and seven females in the MIPO group and 12 males and eight females in the IMN group. In the MIPO group, eight fractures were on the left side and 12 fractures were on the right side. In the IMN group, nine fractures were on the left side and 11 fractures were on the right side. The mean age of the patients in the IMN group was 36.4±13.1 and in the MIPO group was 38.6±15.1 (p-value >0.05). The mean interval from injury to surgery was 15.5±3 days in the MIPO group and 8.15±4.26 in the IMN group (p-value <0.05) (Table 1).

**Table 1:** Demographic parameters and clinical results AOFAS: American Orthopaedic Foot and Ankle Society

Demographic parameter	Plating group	Nailing group	Statistical significance
Age (years)	38.6±15.1	36.4±13.1	>0.05
Left side/Right side	8/12	9/11	>0.05
Male/Female	13/7	12/8	>0.05
Injury surgery interval (days)	12.5±3	13.15±4.26	<0.05
Operative time (minutes)	63.8±10.13	61.2±10.79	>0.05
AOFAS score	91.2±6.81	92.6±5.41	>0.05
Union time (weeks)	21.07±2.05	18.29±2.13	>0.05

The mean operative time in the MIPO group was 63.8±10.13 minutes and in the IMN group was 61.2±10.79 minutes (p-value >0.05). The mean follow-up duration was 14.1±1.74 months in the MIPO group and 14.4±1.63 in the IMN group (P-value >0.05).

The mean union time in this study was 18±2.45 weeks in the IMN group and 20 ± 3.21 in the MIPO group (p-value >0.05).

The mean AOFAS score in the MIPO group was 91.2±6.81 and in the IMN group was 92.6±5.41 (p-value >0.05). The mean union time in the MIPO group was 21.07±2.05 weeks and in the IMN group was 18.29±2.13 weeks.

**Figure 1:**
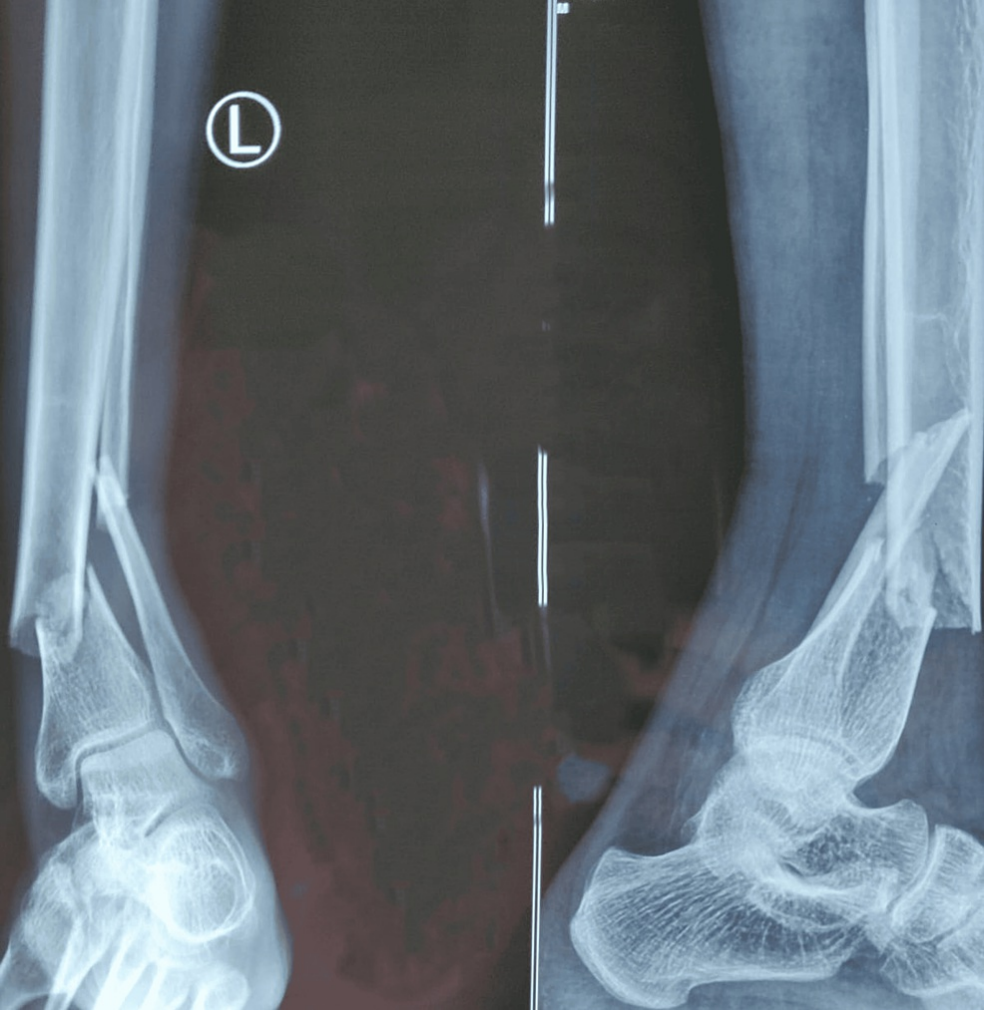
Pre-operative radiograph of distal both bone leg fracture

**Figure 2:**
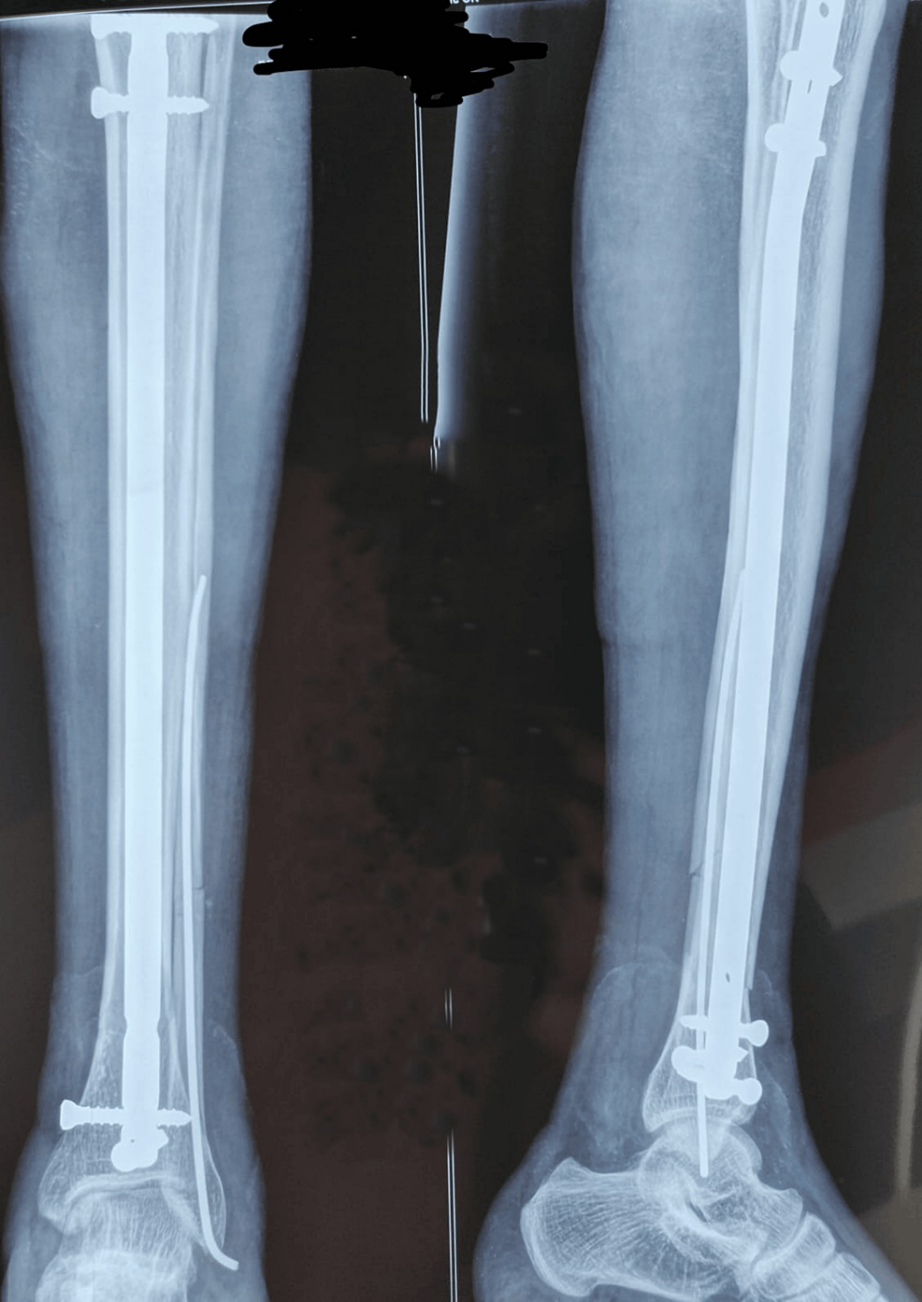
Post-operative radiograph

**Figure 3:**
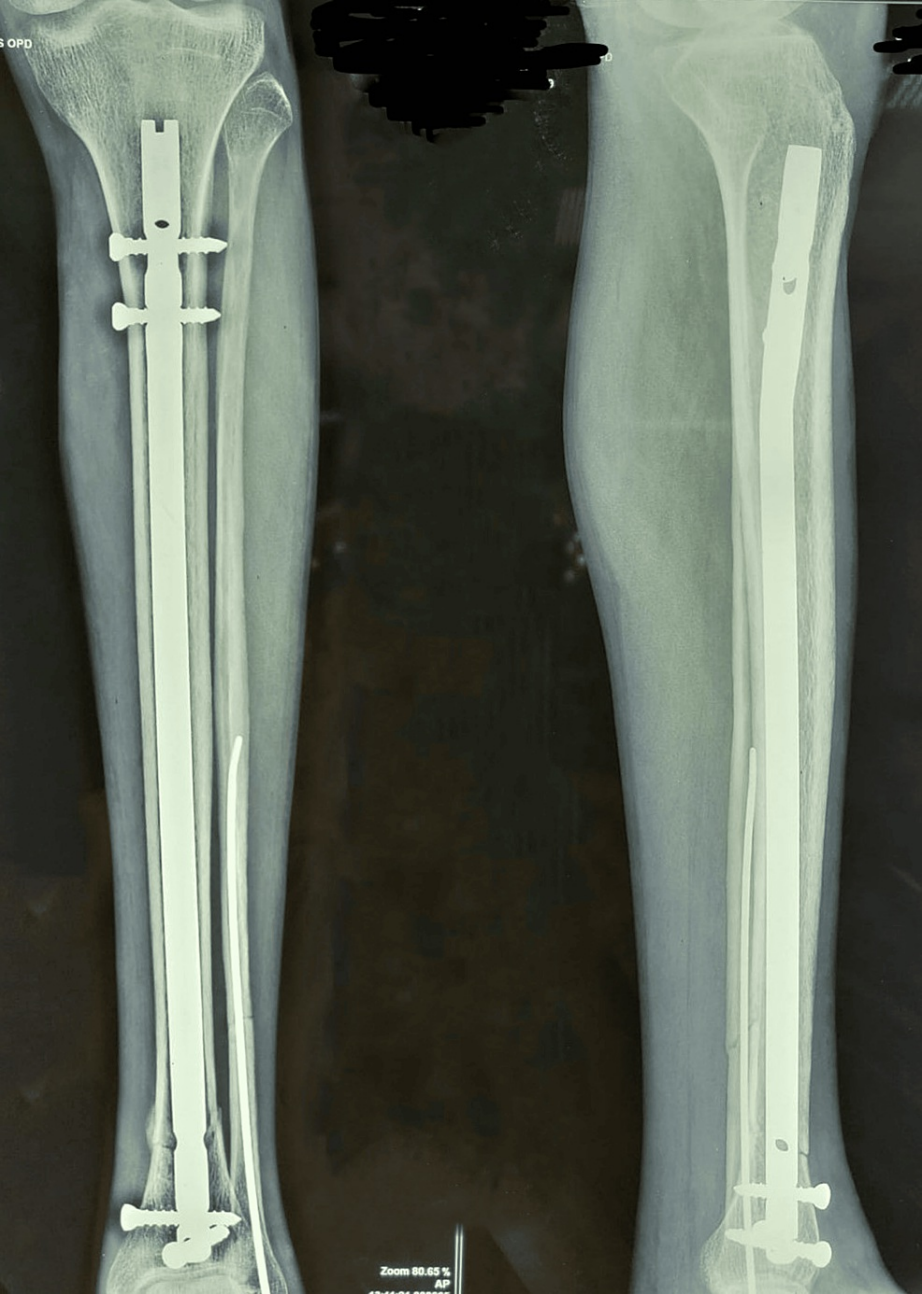
Follow up at six months showing complete union

**Figure 4:**
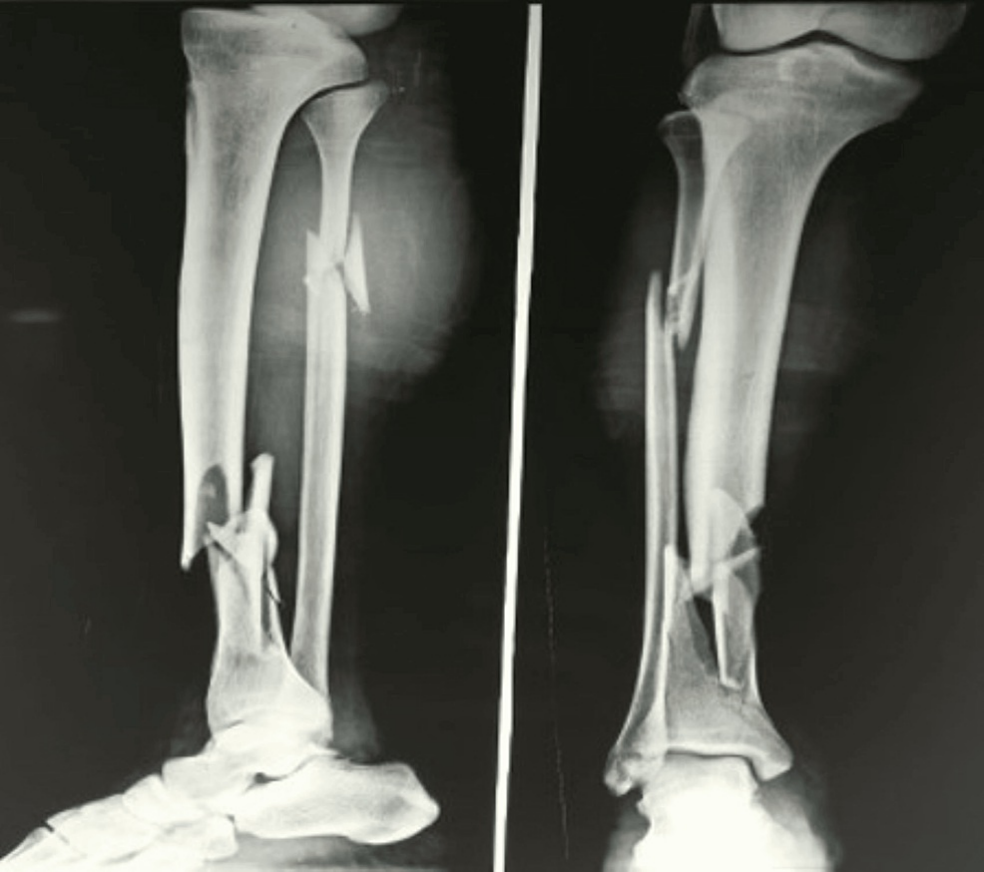
Pre-operative radiograph of distal both bone leg fracture

**Figure 5:**
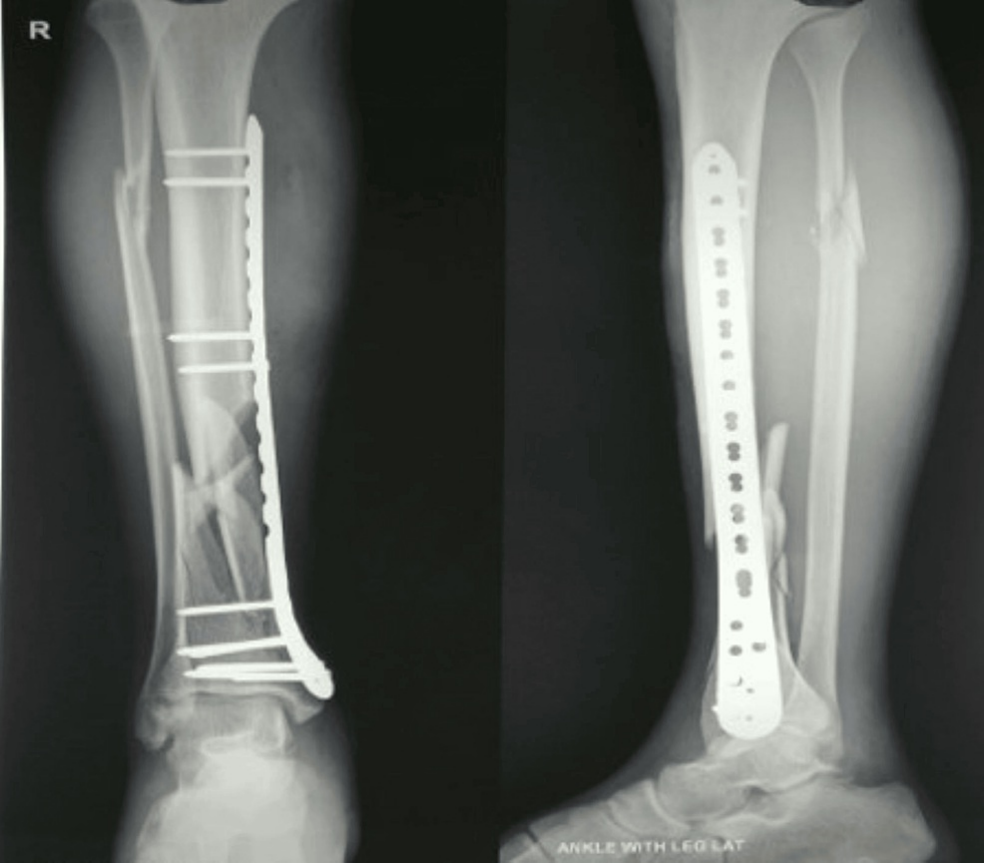
Post-operative radiograph

**Figure 6:**
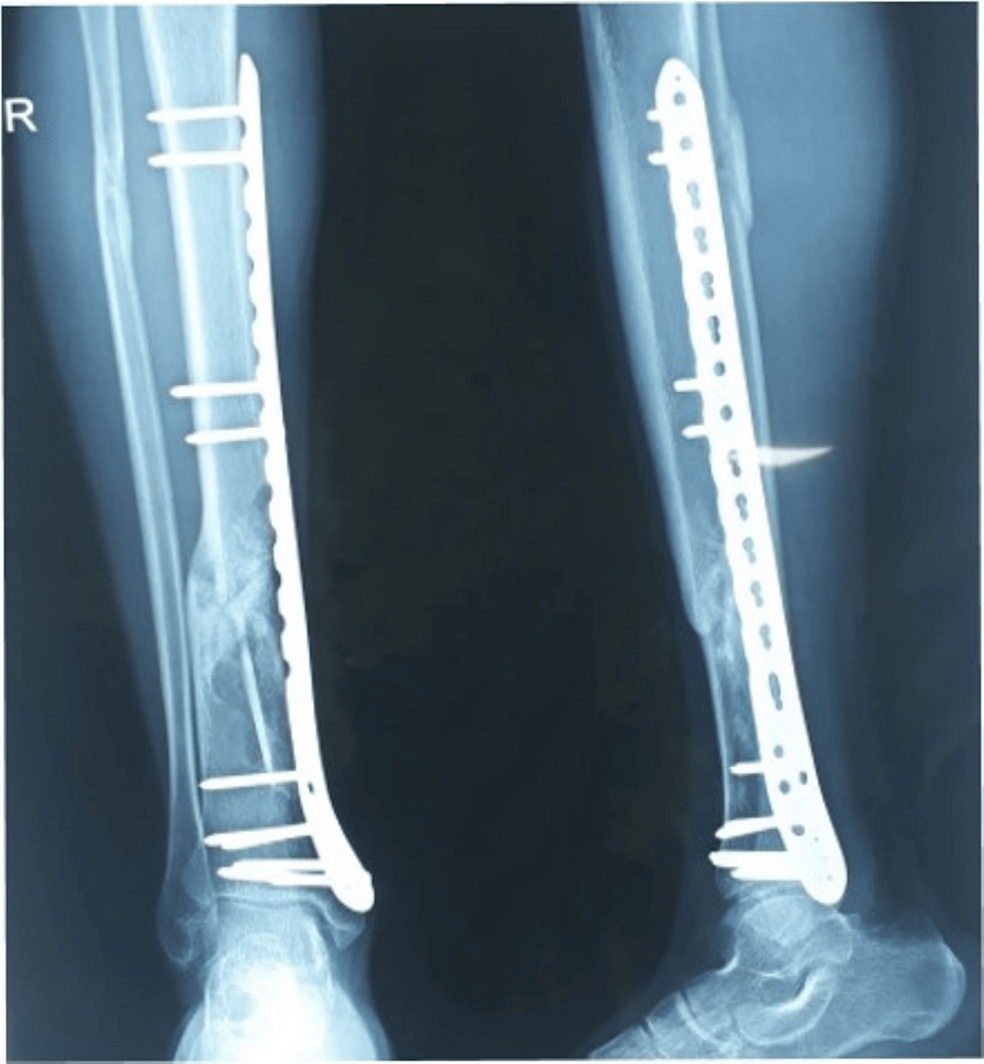
Follow up radiograph at one year showing complete union

Union occurred in 90% (n=18) of patients in the IMN group (Figure 1-3) and 85% (n=17) in the MIPO group (p-value >0.05) (Figure 4-6).

**Figure 7:**
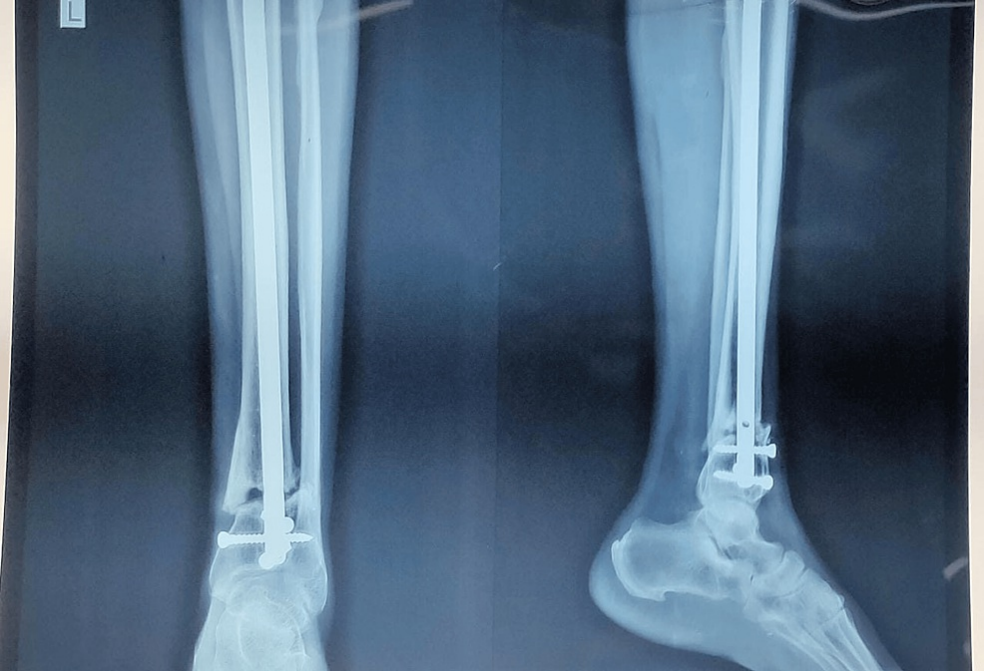
Non-union at distal tibia fracture site treated with intramedullary nailing

Fibula was fixed in 75% (n=15) of patients in the MIPO group and in all the patients of the IMN group. Wound complications and infection occurred in 15% (n=3) patients in the MIPO compared to no infection in the IMN group (p-value <0.05). Non-union occurred in two patients in the IMN group and three patients in the MIPO group (p-value >0.05) (Figure 7).

## Discussion

The treatment of distal tibial fractures has always been considered challenging because of poor blood supply to the skin and bone. In addition, because of the severity of injury, swelling occurs rapidly in the post-injury period, which precludes early internal fixation in these fractures. The goal of treatment in these fractures is to restore length, alignment, and rotation while preserving soft tissue integrity. The long lever arm, canal enlargement in the metaphyseal region, and epiphyseal-metaphyseal fixation problems make reduction and nailing technically difficult in these types of fractures [7,8]. Several studies have reported a high rate of infection and irritation due to the prominence of the implant during MIPO plating of the tibia [9,10].

In this study, 48 patients were enrolled during the study period, of which eight patients were excluded according to the exclusion criteria mentioned. Of the remaining 40 patients, 20 were operated on using the MIPO technique with a distal tibia locking plate. The remaining 20 patients underwent surgery using an intramedullary interlocking nail with a distal tip locking nail and three locking screws in the distal fragment. Fibulae were fixed in 75% (n=15) of patients in the MIPO group at the discretion of the operating surgeon, whereas they were fixed in all patients in the IMN group. Most patients in this study were males 62.5% (n=25). In our study, right-side fractures were more common 57.5% (n=23).

In this study, the mean injury surgery interval was 15.5±3 days in the MIPO group and 8.15±4.26 in the IMN group, which was significant (p value <0.05). This shows that patients can be operated earlier with intramedullary nailing devices than with plating because it is soft tissue friendly. In a study by Vidovic et al. [11] on distal tibial MIPO plating, the authors reported a mean time to operation of 10.4 days (range 7-14 days) in patients in whom an external fixator was applied.

In our study, the mean operative time in the MIPO group was 63.8±10.13 minutes and in the IMN group it was 61.2±10.79 min (p-value >0.05). In the study done by Daolagupu et al. [12], Guo et al. [13], and Janssen et al. [14], significantly longer operative time in the locking compression plate (LCP) group compared with the IMN group was reported (p-value <0.001).

Studies conducted by Yang et al. [15] and Dolagapu et al. showed earlier union in the IMN group, which was significant. Other studies such as Guo et al., Ram et al. [16], Im et al. [17], and Janssen et al. found no significant difference in the union rate between the plating and IMN groups. We found a shorter union time in the IMN group than in the MIPO group, but the difference was not statistically significant (p-value<0.05).

The mean AOFAS scores in the MIPO and IMN groups were comparable in our study (p-value >0.05). Guo et al. and Patil et al. [18] also reported comparable scores between the two groups. Daolagupu et al. also showed similar functional outcomes between the two groups, but using different functional outcome criteria (Johner and Wruh’s criteria).

In our study, no infection or wound complications occurred in the IMN group, whereas these complications occurred in 15% of cases (n=3) in the MIPO group. There was no significant difference (p-value 0.42) between the nailing and plating groups in Vallier et al. [19]. Daolagupu et al. reported higher rates of infection and implant irritation in the plating group than in the nailing group, which is comparable to our results.

A limitation of our study is that it is a non-randomised study, which may result in bias. Another limitation is the shorter follow-up and lesser number of subjects in this study, which may be the cause of the insignificant difference in functional outcome. Fibula fixation, on the one hand, improves alignment; on the other hand, it may result in delayed union and non-union. Addressing this issue will require a specific randomised controlled trial on a larger number of subjects comparing outcomes between fibula fixation and non-fixation groups.

## Conclusions

We conclude that both intramedullary nailing and MIPO plating produce satisfactory results for the treatment of distal tibial fractures. With regard to union time and union rate, both treatment methods showed no statistically significant difference. MIPO for distal tibial fracture results in a higher rate of wound complications than intramedullary nailing.

## References

[1] Bucholz R, Court-Brown C, Rockwood C (2015). Rockwood and Green’s Fracture in Adults. 8th-ed. https://www.worldcat.org/title/Rockwood-and-Green's-fractures-in-adults/oclc/878915244.

[2] Müller M, Nazarian S, Koch P, Schatzker J (1990). The Comprehensive Classification of Long Bones. The Comprehensive Classification of long bones.

[3] Duda GN, Mandruzzato F, Heller M, Goldhahn J, Moser R, Hehli M (2001). Mechanical boundary conditions of fracture healing: borderline indications in the treatment of unreamed tibial nailing. J Biomech.

[4] Habernek H, Kwasny O, Schmid L, Ortner F (1992). Complications of interlocking nailing for lower leg fractures: a 3-year follow up of 102 cases. J Trauma.

[5] Borrelli J Jr, Prickett W, Song E, Becker D, Ricci W (2002). Extraosseous blood supply of the tibia and the effects of different plating techniques: a human cadaveric study. J Orthop Trauma.

[6] Rodrigues RC, Masiero D, Mizusaki JM (2008). Translation, cultural adaptation and validity of the American orthopaedic foot and ankle society (AOFAS) ankle-hindfoot scale. Acta Ortop Bras.

[7] Hahn D, Bradbury N, Hartely R, Radford PJ (1996). Intramedullary nail breakage in distal fractures of the tibia. Injury.

[8] Nork SE, Schwartz AK, Agel J, Holt SK, Schrick JL, Winquist RA (2005). Intramedullary nailing of distal metaphyseal tibial fractures. J Bone Joint Surg Am.

[9] Bourne RB (1989). Pylon fractures of the distal tibia. Clin Orthop Relat Res.

[10] Bhattacharyya T, Crichlow R, Gobezie R, Kim E, Vrahas MS (2006). Complications associated with the posterolateral approach for pilon fractures. J Orthop Trauma.

[11] Vidović D, Matejčić A, Ivica M, Jurišić D, Elabjer E, Bakota B (2015). Minimally-invasive plate osteosynthesis in distal tibial fractures: results and complications. Injury.

[12] Daolagupu AK, Mudgal A, Agarwala V, Dutta KK (2017). A comparative study of intramedullary interlocking nailing and minimally invasive plate osteosynthesis in extra articular distal tibial fractures. Indian J Orthop.

[13] Guo JJ, Tang N, Yang HL, Tang TS (2010). A prospective, randomised trial comparing closed intramedullary nailing with percutaneous plating in the treatment of distal metaphyseal fractures of the tibia. J Bone Joint Surg Br.

[14] Janssen KW, Biert J, van Kampen A (2007). Treatment of distal tibial fractures: plate versus nail: a retrospective outcome analysis of matched pairs of patients. Int Orthop.

[15] Yang SW, Tzeng HM, Chou YJ, Teng HP, Liu HH, Wong CY (2006). Treatment of distal tibial metaphyseal fractures: plating versus shortened intramedullary nailing. Injury.

[16] Ram GG, Kumar D, Phagal VV (2014). Surgical dilemma’s in treating distal third leg fractures. Int Surg J.

[17] Im GI, Tae SK (2005). Distal metaphyseal fractures of tibia: a prospective randomized trial of closed reduction and intramedullary nail versus open reduction and plate and screws fixation. J Trauma.

[18] Patil R, Gowaikar A, Shirke A (2020). A comparative study of extra-articular distal tibia fractures managed by intramedullary nailing vs locking plate. Int J Orthop Sci.

[19] Vallier HA, Cureton BA, Patterson BM (2011). Randomized, prospective comparison of plate versus intramedullary nail fixation for distal tibia shaft fractures. J Orthop Trauma.

